# Comparison of the Effect of Tanacethum Parthenium, 5-Hydroxy Tryptophan, and Magnesium (Aurastop) versus Magnesium Alone on Aura Phenomenon and Its Evolution

**DOI:** 10.1155/2019/6320163

**Published:** 2019-10-09

**Authors:** Giorgio Dalla Volta, Paola Zavarise, Laura Perego, Lidia Savi, Alessandro Pezzini

**Affiliations:** ^1^Headache Center, U.O Neurologia, Istituto Clinico Citta' di Brescia, Brescia, Italy; ^2^Headache Center, Azienda Ospedaliera Univesitaria Citta' Della Salute e Delle Scienze di Torino, Turin, Italy; ^3^Dipartimento di Scienze Cliniche e Sperimentali, Clinica Neurologica, Università Degli Studi di Brescia, Brescia, Italy

## Abstract

None of the clinical trials on migraine conducted thus far have focused on the possibility to modulate the phenomenon of aura. Furthermore, whether proper management of aura results in a better control of the headache phase has been poorly investigated. In the setting of a single-center, pilot, clinical trial, we aimed at comparing the effects of Aurastop (a combination of tanacetum parthenium (150 mg extracted at 0.8% = 1.2 mg di of active parthenolide), griffonia simplicifoila (20 mg of 5-hydroxy tryptophan), and magnesium (185 mg of magnesium pidolatum)) with those of magnesium alone (2.25 grams/tablet, corresponding to 184 mg of Mg++) in the treatment of acute attacks of migraine with aura. Between June 2017 and June 2018, 50 consecutive patients (27/23 male/female; mean age, 31 [18–57] years) with at least 3 episodes of aura per year were included (*t*_0_). Participants were instructed to keep track of the following 4 episodes of migraine with aura (*t*_1_) and invited to assume (1) a tablet of Aurastop at the beginning of the following 2 episodes of aura and (2) a magnesium tablet alone at the occurrence of the third and fourth aura attacks. Forty-eight patients (96.0%) had >50% reduction in aura duration when treated with Aurastop vs. 7 patients (14.0%) when treated with magnesium alone (*p* < 0.001); 48 patients (96.0%) had >50% reduction of aura-related disability when receiving Aurastop vs. 5 patients (10.0%) when treated with magnesium alone (*p* < 0.001); however, patients receiving Aurastop did not need to take pain killers in 35% of aura attacks vs. 3% when assuming magnesium (*p* < 0.001). These results support the hypothesis that Aurastop might be effective in interfering with the phenomenon of aura and provide evidence that the clinical benefit attributable to this combination of molecules might be greater than that obtained with single compounds of proven effect on the biology of migraine.

## 1. Introduction

In about 25% of people suffering episodic migraine, the headache phase is preceded by the aura (migraine with aura), which is traditionally considered a nontreatable phenomenon [1]. The clinical trials conducted thus far have focused on the headache phase and the possibility to modify pain duration and severity with migraine-specific compounds. None of such trials, however, have been specifically designed with the aim of interfering with and modulating the process of cortical spreading depression (CSD) in its early stage, before pain occurrence [2]. At least theoretically, a very early intervention that is successful in blocking the aura phenomenon could result not only in the modification of the clinical features of aura itself but also in a better control of headache. Recently, we reported the results of a retrospective, observational study, emphasizing the beneficial effect of the new compound Aurastop (a combination of tanacetum parthenium, 5-hydroxy tryptophan, and magnesium) in reducing aura duration and aura-related degree of disability when assumed at the very beginning of aura in the majority of patients [3]. The hypothesis that such a clinical benefit is likely due to the effect of Aurastop on the biochemical phenomenon of CSD is indirectly supported by our observation of a reduction in postaura headache, as well as pain intensity and duration, and increase in sensitivity to the effect of common analgesics/triptans in patients taking this new drug. These results were confirmed by a further multicentric observational study conceived by the collaboration of 9 different Headache Centers in Italy on a cohort of 200 patients suffering migraine with aura [4]. Based on these premises, in the present study, we aimed at confirming those preliminary results and ruling out any theoretical placebo effect, by comparing the effects of Aurastop with those of magnesium alone (assumed at the same dosage as in the Aurastop) in the treatment of acute attacks of migraine with aura.

## 2. Patients and Methods

Patients eligible for the present study were selected among those referred to the Headache Centre of the Istituto Clinico Città di Brescia, Brescia, Italy, between June 2017 and June 2018. Each patient underwent a detailed clinical and neurological examination. The diagnosis of migraine with aura was made by experienced headache specialists, according to the International Classification of Headache Disorders (ICHD)-3 beta criteria [5].

The following inclusion criteria were considered:

1. Age between 18 and 55 years
2. At least 3 episodes of aura per year, with a minimum aura duration of 20 minutes.

Patients did not qualify for the study and were, therefore, excluded if they were assuming migraine preventive treatment since at least 6 months at the time of clinical evaluation. Aurastop®, proposed as supplement, was a combination of the 3 molecules tanacetum parthenium (150 mg extracted at 0.8% = 1.2 mg di of active parthenolide), griffonia simplicifoila (20 mg of 5-hydroxy tryptophan), and magnesium (185 mg of magnesium pidolatum). Magnesium was proposed as a 2.25 gram tablet (corresponding to 184 mg of Mg++).

### 2.1. Study Design

At baseline (*t*_0_), we retrospectively characterized the natural history of the aura phenomenology. In this regard, patients included in the study received a migraine headache diary on which they were instructed to report usual (1) aura subtype (only visual or somatosensory; visual and somatosensory; visual, somatosensory, and speech/language symptoms (here defined as complex)), (2) aura duration (in minutes), (3) aura-related disability (on a scale ranging from 0 (no disability) to 5 (maximal disability)), (3) presence of concomitant/following headache characteristics (duration, intensity (on Visual Analogue Scale, 0 to 10)), and (4) eventual use of pain killers (triptans, nonsteroidal anti-inflammatory drugs) with a personal evaluation of their efficacy (response to symptomatic therapy), before the study began. Patients were also invited to keep track of the following 4 episodes of migraine with aura. Each patient received a blister with 4 tablets of Aurastop®, with the instruction to assume a tablet at the beginning of the following 2 episodes of aura, recording aura duration and disability on headache diary, and a second tablet at the beginning of pain (in case pain occurred). Patient was allowed to take the usual pain killer after 1 hour if the pain was persistent. Similarly, each patient received a blister of 4 magnesium tablets to be taken at the occurrence of the third and fourth aura attacks following the same protocol. We then further evaluated patients and migraine headache diaries after these 4 aura episodes (*t*_1_).

### 2.2. Outcome Measures

Primary end points were the >50% reduction in duration and disability of the aura phenomena. Number of headache attacks, use of analgesics, and response to symptomatic treatment were considered as secondary end points.

This study is an audit of outcome, and as such, it does not require ethics committee approval according to the Italian rules.

### 2.3. Statistical Analyses

Outcome end points in the two groups of treatment were compared by *χ*^2^ analysis. *p* ≤ 0.05 on two-sided test was considered significant. Data were analyzed using the SPSS (version 21.0) package (http://www.spss.com).

## 3. Results

Fifty patients (27/23 males/females; mean age, 31 [18–57] years) were qualified for the analysis. The pretreatment characteristics of migraine aura in these patients are presented in Table 1. Overall, as expected from epidemiologic data and in line with the ICHD-3 beta criteria [5], all the aura episodes (*n* = 150) had visual manifestations (negative scotoma or fortification spectrum), although somatosensory (pins and needles moving slowly from one hand to the face) and complex phenomena (different types of aura following one another in succession) were reported in a minority of patients.

**Figure 1:**
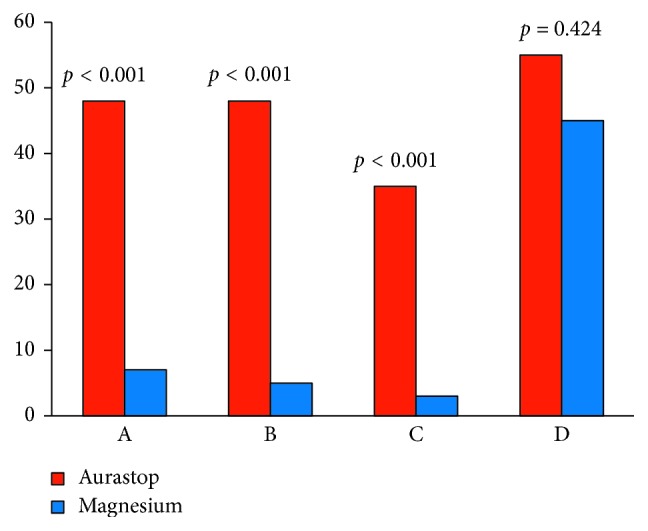
Histogram of the comparison of effects of Aurastop and magnesium on the number of patients who experienced >50% reduction in aura duration (A), aura-related disability (B), did not need to take pain killers (C), and percentage of patients who experienced increased benefit from pain killers (D).

Mean aura duration was quantified in 46.1 ± 16.3 minutes. The effects of Aurastop and magnesium alone on the characteristics of migraine aura are summarized in Figure 1. Overall, 48 patients (96.0%) reached the primary end point of >50% reduction in aura duration when treated with Aurastop (*t*_0_ = 46.1 ± 16.3 minutes vs*. t*_1_ = 15.2 ± 11.2 minutes; *p* < 0.01) versus 7 (14.0%) when treated with magnesium alone (*p* < 0.001; Figure 1(A)). Similarly, >50% reduction in aura-related disability was observed in 48 patients (96.0%) when receiving Aurastop versus 5 patients (10.0%) when treated with magnesium alone (*p* < 0.001; Figure 1(B)). As to the secondary end point, patients receiving Aurastop did not need to take pain killers in 35% of aura attacks versus 3% when assuming magnesium (*p* < 0.001; Figure 1(C)), while 27 patients (54.0%) reported increased benefit from pain killers when taking Aurastop versus 22 (44.0%) when assuming magnesium (*p* = 0.424; Figure 1(D)).

No major side effects were detected with both Aurastop and magnesium.

## 4. Discussion

The main finding of the present study is the observation that the majority of patients (96.0%) receiving the new combination of molecules Aurastop had a significant reduction in aura duration and aura-related disability in comparison with the usual characteristics of aura before the assumption of this compound and with the effects obtained with the use of magnesium alone. Similarly, Aurastop showed a relevant impact in reducing the number of patients who experienced a headache phase after aura, as well an in increasing the analgesic effect of pain killer molecules.

These findings, in line with those previously obtained by Zavarise et al. and Dalla Volta et al. [3, 4], implicate the possibility that the new drug might interfere with neuronal hyperexcitability and the phenomenon of CSD, as well as with the synaptic transmission mediated by TRPA1 receptors implicated in the release of CGRP from the perivascular terminals of neurons involved in the neurogenic inflammation (a specific effect of parthenolide, the active metabolite of tanacetum parthenium [6, 7]), and by NMDA glutamatergic channels activity, inhibited by the specific effect of 5-hydroxy tryptophan [8, 9] through the kynurenine pathway activation. From a clinical point of view, our data support the hypothesis that some molecules may modulate the aura phenomenon, as well as the subsequent headache phase and accompanying symptoms, and also confirm the potentials of the new compound Aurastop in this regard. In particular, among the 3 components of Aurastop, tanacetum parthenium, because of its kinetics (elution time, 1.33 minutes; absorption, less than 3 minutes; rapid crossing of the blood-brain barrier [10, 11]), is probably the most efficient in influencing the cortical electric phenomena leading to CSD. Moreover, the kynurenic acid derived from the metabolism of L-tryptophan, acts as endogenous antagonist of NMDA receptors. Increased serum concentrations of L-tryptophan, such as those obtained taking the new compound Aurastop, have, therefore, a direct influence on the kynurenic pathway, which, in turn, leads to both an antagonistic effect on the hyperactivation of the peripheral NMDA receptors and the trigeminal-vascular system and the development of CSD and changes in BBB permeability.

There are some methodological aspects of our study that are worth to be noted. The reason why we decided to use magnesium alone as comparator in this head-to-head comparison is because of its well-known effect in the regulation of neuronal excitability [12] and also because of its inhibitory effect of NMDA receptor, which, in turn, is involved in the excitatory glutamatergic activity leading to neurogenic inflammation, central sensitization, and CSD. Since magnesium is an active compound with a proven effect on migraine, any theoretical placebo effect underlying previous results obtained with the therapeutic use of Aurastop is, at least, significantly reduced if not definitively ruled out. There are also some limitations of our analysis worthy of consideration. First, the number of patients included in this pilot study is relatively small, and findings should be confirmed in a larger cohort of patients with migraine with aura, ideally in the setting of a randomized case-control trial. Second, at least theoretically, we cannot exclude that comorbidities or other clinical variables not included in the analysis might have had an influence on the response to treatment. However, since each patient assumed both the two treatments under investigation, although at different points in time, it seems most unlikely that the results might be explained by different natural history of aura or by a differential effect of covariates in the same individual. Obviously, also from this point of view, a randomized control trial would be the preferred methodological approach to compare the clinical effects of the two compounds.

## 5. Conclusion

The results of the present analysis not only support the hypothesis that Aurastop might be effective in interfering with the phenomenon of aura, probably because of its effects on the underlying CSD, but also, more important from a clinical point of view, they provide a first evidence that the clinical benefit attributable to this combination of molecules is greater than that obtained with a single compound of proven effect on the biology of migraine. The theoretical possibility that these effects are due to a placebo effect seems, therefore, unlikely.

## Figures and Tables

**Table 1:** Baseline demographic characteristics and pretreatment/posttreatment features of the 150 aura episodes detected in the study cohort.

	Pretreatment migraine aura *n* (%)	Posttreatment migraine aura *n* (%)	*p*-value
Sex, F	23 (46)		
Age, years, mean ± SD	31 ± 12		
Family history of any migraine	35 (70)		
Family history of migraine with aura	5 (10)		
Aura characteristics			
Visual	150 (100)	150 (100)	ns
Sensory	35 (23.3)	25 (16.6)	0.002^*∗*^
Complex	2 (1.3)	1 (0.0)	ns
Duration, minutes, mean ± SD	46.1 ± 16.3		
Migraine age of onset, years, mean ± SD	18 ± 2		
Migraine without aura	34 (68)		

^*∗*^Variables were compared by McNemar's *χ*^2^ analysis.

## Data Availability

The data used to support the findings of this study are available from the corresponding author upon request.
